# HIV Pre-exposure Prophylaxis in the LGBTQ Community: A Review of Practice and Places

**DOI:** 10.7759/cureus.15518

**Published:** 2021-06-08

**Authors:** Jennifer Dorcé-Medard DO, Okelue E Okobi MD, Jesse Grieb DO, Nzingha Saunders DO, Seneca Harberger MD

**Affiliations:** 1 Family Medicine, Lakeside Medical Center, Belle Glade, USA

**Keywords:** pre-exposure prophylaxis, hiv, men who have sex with other men (msm), bisexual, transgender, acquired immune deficiency syndrome (aids), homosexual

## Abstract

One in six bisexual and gay men will be diagnosed with HIV in their lifetime (Human Rights Campaign, 2017). Without a vaccine or cure, prevention may be the best tool to control the HIV pandemic. Since 2012, the World Health Organization (WHO) recommends HIV pre-exposure prophylaxis (PrEP) for a high-risk population. To this day, in the United States (U.S.), the group with the highest risk is MSM (men who have sex with other men) that have condom-less sexual intercourse (Center for Disease Control and Prevention, 2019). In fact, in 2018, over 50% of all HIV infections occurred in MSM and members of the LGBT community.

A systematic review was conducted using PubMed, Google Scholar, and Cochrane Library. The inclusive criteria were articles published from 2015-2020, focusing specifically on HIV PrEP among the members of the LGBTQ+ community. The keywords for our search were "Pre-exposure prophylaxis," "HIV," "men who have sex with other men" (MSM), "Bisexual," "transgender."

A total of 25 articles met the inclusion criteria. About 50% of the articles focused on MSM while others highlighted bisexual and transgender women. Globally, PrEP is a priority. Programs to educate and promote its use are being developed, but challenges are present regarding access to PrEP and its maintenance for longer than 12 months. In the U.S., PrEP programs started in 2012, intending to promote and educate. Research shows that more than 60% of the high-risk patients are willing to try PrEP if they are correctly educated, showing that physicians were not doing a thorough job educating their patients.

PrEP is essential for preventing the transmission of HIV among the LGBTQ+ subpopulation. Existing gaps need to be bridged to create or improve to educate high-risk populations and physicians on HIV PrEP.

## Introduction and background

One in six bisexual and gay men will be diagnosed with HIV in their lifetime [1], without a vaccine or cure, prevention may be the best tool to control the HIV pandemic. Since 2012, the World Health Organization recommends HIV pre-exposure prophylaxis (PrEP) for a high-risk population. To this day, in the United States (U.S.), the group with the highest risk are men sleeping with med (MSM) that has condom-less sexual intercourse [2]. In fact, in 2018, over 50% of all HIV infections occurred in MSM and members of the LGBT community.

Approximately two million people became infected with HIV in 2015, demonstrating the dire need for effective, safe, and accessible prevention options. In 2012, the World Health Organization (WHO) made its first recommendation for the use of HIV pre-exposure prophylaxis (PrEP) for men who have sex with men and serodiscordant couples [3]. In 2015, WHO recommended offering PrEP to all persons at substantial risk of HIV infection [4]. PrEP is a preventative option for people who do not have HIV but are at high risk of getting the infection. This prophylaxis involves using a once-daily pill of emtricitabine (FCT)-tenofovir disoproxil fumarate (TDS). This has been demonstrated to be highly efficacious at preventing the acquisition of HIV in people at risk from different sexual exposure methods [5]. Studies conducted in different countries show that adherence to HIV treatment varies considerably [6]. FTC-TDF is a safe treatment with minimal side effects. Access to PrEP continues to be a challenge in many parts of the world; the main reason has been attributed to limited knowledge among the high-risk groups, but cost plays a significant role [7]. The Joint United Nations Program on HIV/AIDS and WHO has made PrEP implementation a priority in at-risk populations [8]. Though it is available in the U.S., the numbers are still deficient compared to what they could be for individuals identifying as LGBTQ+, identified as a high-risk group, PrEP is critical. Most HIV infections (almost 50%) occur in MSM. Therefore, prevention in this group is key [9]. Interestingly, biological and transgender women represent a challenge since they reach lower blood concentrations of FCT-TDF due to blood estrogen levels, making it harder to prevent HIV infection. More studies are necessary to determine the possible repercussions [10]. Studies show that most members of the LGBT community do not know about the benefits of PrEP when asked, but up to 70% of them claimed that they might try it if given a chance. In this review, we believe it will be vital to review the current trends on pre-exposure prophylaxis for HIV among the LGBTQ+ community on effective practices in the U.S. and worldwide.

Inclusion criteria

The following were included: 1. Randomized clinical trials, systematic reviews, observational studies, and meta-analysis publications on PrEP between 2015 -2021, and 2. Government policies and recommendations on PrEP

Exclusion criteria

The following were excluded: 1. Published articles on PrEP before 2015, 2. Commentaries and traditional news media reports, 3. Articles that included other populations such as injection drug users, serodiscordant couples, and straight sexual workers, 4. Articles that target other kinds of prophylaxis different from oral FTC-TDF, 5. Articles that target the low-risk group, lesbians.

After our inclusion and exclusion criteria were reviewed, we found 32 articles and excluded seven, finally reviewing 25 articles in our submission.

Methodology

Our objective is to determine the current trends involving HIV PrEP among the LGBT community in the last five years. We conducted a systematic review using PubMed, Google Scholar, and Cochrane Library. The inclusive criteria were articles published between 2015 and 2020. They focused specifically on pre-exposure prophylaxis of HIV in any of the members of the LGBTQ+ community (Gay, Lesbians, Bisexual, Transgender, Questioning, and Queer). Articles referring to programs or guidelines for PrEP among the LGBT community were taken into consideration.

The keywords for our search were "Pre-exposure prophylaxis," "HIV," "men who have sex with other men" (MSM), "bisexual," "transgender." Articles that included other populations such as injection drug users, serodiscordant couples, and straight sexual workers were excluded, and articles that target other kinds of prophylaxis different from oral FTC-TDF. Lesbians were not included since the risk of acquiring HIV infection in women who have sex with women is uncommon; thus, they were not included among the high-risk groups and did not qualify for PrEP. This resulted in only 25 selected articles meeting the criteria for this review. Each article was selected using a two-step process, screening titles and abstract first and assessing the full text of records included after. Key findings were extracted corresponding to the central questions of our review. Upon reviewing each article, the information was divided by groups taking regions and gender into consideration. The results' discussion mainly was focused on PrEP implementation and trends worldwide, with a particular focus on the United States. Also, we decided to include articles that presented relevant information comparable to the population at our clinic in Florida.

## Review

Results/Discussion

After applying our inclusion and exclusion criteria, a total of 25 articles made it to further review. About 50% were articles referring specifically to MSM. The remaining were focused on bisexual and transgender women. HIV transmission among MSM remains a challenge and reducing the HIV disease burden in this subpopulation is key to ending the HIV pandemic [2].

Men who have sex with other men (MSM) and who engage in condom-less anal intercourse with casual partners are at high risk of acquiring sexually transmitted infections, including HIV [11]. The risk of contracting HIV among this population is 27 times higher than in any other demographics. In North America, cases of HIV among MSM represent over 50% of all cases [12]. Transgender women face an added challenge; studies show that reaching an adequate blood concentration of PrEP is difficult among this subgroup [10].

The first study exploring the real-world effectiveness of daily oral PrEP among MSM was the PROUD study (Pre-exposure Option for reducing HIV in the UK: immediate or Deferred) [13]. This phase 3, randomized open-label waitlisted trial was conducted on HIV-negative MSM who had anal intercourse without a condom in the previous 90 days. Two groups were set, and participants were randomly assigned to start prophylaxis with FTC-TDF immediately or after the deferral period of 12 months. Six months after the start of the trial, the deferred group participants were offered PrEP. When comparing results, three out of 259 participants in the immediate group were positive for HIV while 20 out of 245 participants were positive in the deferred group. The study concluded that daily FTC-TDF conferred higher protection against HIV than placebo, confirming previous results obtained in placebo-controlled trials.

Another study evaluated on-demand prophylaxis in MSM for HIV infection by conducting a double-blind, randomized trial IPERGAY (Intervention Prophylactique pour Et avec les Gays) [14]. Participants were randomly assigned to take FTC-TDF or a placebo before and after sexual activity. Patients also received counseling and condoms and were tested for HIV and other sexually transmitted infections. A total of 400 participants HIV negative were included in the study; 16 HIV infections occurred during follow-up, two from the FTC-TDF group and the rest from the placebo group. In conclusion, the use of TDF-FTC before and after sexual activity protected about 86% of MSM.

There are various programs around the world trying to increase the PrEP for HIV in MSM. For instance, the Public Health Agency of Sweden recommends PrEP for high-risk HIV individuals, particularly men who have sex with men since 2017. A study evaluated the introduction of PrEP to prevent HIV among MSM and provided insights using a mathematical pair formation model [15]. The study divided the participants based on sexual partners and casual sex encounters, and sexually active MSM and sexually active MSM. By dividing the population into these two groups, the study proved that achieving PrEP coverage of at least 3.5% among the highly sexually active MSM will reduce HIV infection to 0%. In contrast, evaluating the low sexually low activity group showed that coverage of 34.4% of the population would be needed to reduce the incidence of HIV infection to 0%. The study concluded that even though models that include different stages of HIV infection and real-world data are needed, targeting the high-risk individuals with high sexual activity seems to be the key to prevent HIV infections effectively.

PrEP has been available in England since October of 2017, and it is recommended for high-risk HIV-negative populations, including MSM and transgender women. A study evaluated PrEP knowledge and used UK MSM and transgender women changes from 2013-2018 [16]. The study evaluated the attitudes and understanding of PrEP in MSM recruited from three sexual clinics in England; HIV negative and over 18 years old. Participants completed a questionnaire in the clinic and were asked to subsequently complete four-monthly and annual questionnaires. The team assessed the trends over calendar time, three months from first enrollment to the end of the period (six months). The study observed a substantial increase in PrEP awareness among MSM men from 2013 to 2018 in England. As a recommendation, the authors suggest that improving access to PrEP by routine commissioning could increase PrEP use among eligible MSM.

In Canada, MSM accounts for approximately half of new HIV infections. In 2017, the use of PrEP was approved, and promotion started. The recommendations are to use PrEP on HIV-negative patients with a high risk of infection daily by taking FCT-TDF pills. As part of their high-risk population, MSM was included. The recommendations also suggest using on-demand PrEP only on MSM [17]. Studies show that to get PrEP in Canada, this needs to be prescribed by a doctor who is willing to provide the necessary follow-up safely. However, there were limited physicians knowledgeable about PrEP thus reducing the number able to provide this service. A research study evaluated knowledge about PrEP both in HIV-positive and HIV-negative MSM through an interview [18]. Less than 30% of the participants were aware that PrEP as an option in both groups, but none had used it before.

Australia has established PrEP trials and education to increase its use among MSM and bisexual men. The Following Lives Undergoing Change Study (FLUX) is a national, online open-prospective observational study among gay and bisexual men [19]. The participants were asked to respond to baseline and six-monthly follow-up questionnaires. The results show that among gay and bisexual men who met the criteria for the study, 69.8% did not commence PrEP. The most commonly associated factors were younger age, living in Australia without PrEP trials, and lower social engagement with gay men. The study concluded that despite meeting formal criteria for PrPE, men who were less sexually active were less likely to initiate PrEP.

In lower-income countries, accessibility and knowledge of PrEP are significantly lower. A review selected 23 studies that evaluated PrEP awareness and willingness in lower and middle-income countries, finding that the proportion of MSM aware of PrEP was less than 30%; however, evaluating willingness was higher at 64% [20]. Overall, deficiencies in knowledge, doubt about effectiveness, fear of side effects, and low perception of HIV risk were the main findings. The study also found how anticipated stigma from peers, partners, and family members related to sexual orientation were potential barriers for starting and adhering to PrEP. Another study in Thailand evaluated the non-adherence of MSM and transgender to PrEP treatments [21]; the results show 37.4% of participants showed low adherence to the PrEP schedule, and the main factors associated were younger age and being transgender. Another study in Thailand evaluated 1467 MSM and 230 transgender women who started PrEP [22]. Forty percent (40%) of them had had condomless anal relationships in the past three months. When evaluating the retention to PrEP at months one, three, six, nine, and 12, retention decreased progressively from 74.2% on month one to 43.9% by month 12. Low levels of education and being young were related to non-adherence to the treatment. The study concluded that although patients are willing to start PrEP programs, innovative retention solutions are needed.

In Myanmar, the willingness to use HIV PrEP among gay men and transgender women was evaluated [23]. Gay men and transgender women were recruited through local prevention outreach programs in 2015. Quantitative surveys were administered, and data on demographic, sexual risk testing history, and PrEP acceptability were collected. Among the 434 participants, awareness was only 5%, but acceptability was high (62%). The study divided the participants depending on who perceived themselves as likely to become HIV positive, who had more than one regular partner, had no regular partners, and had five or more casual sexual partners, showing that the participants with higher numbers of casual partners were more willing to starting PrEP. In conclusion, the study showed that if adequately explained, Myanmar gay and transgender women are likely to initiate PrEP.

In Zimbabwe, a qualitative study was conducted to explore patient motivation to take or decline PrEP and to continue or decline PrEP. One-hundred fifty (150) HIV-negative clients screened as being at high risk of HIV were offered PrEP for six months. In total, 55 people accepted to participate after an interview and receiving education. The results show that PrEP uptake was driven by risk perception of HIV, mainly by the partner's unsafe behavior or HIV-positive status. It is essential to state that after starting PrEP, participants reported a decreased use of condoms, raising a concern since the use of condoms prevents other sexually transmitted illnesses. Zimbabwean authorities recognize that HIV infection is a public health issue that affects the LGBT community and straight men and women. When analyzing the possible causes for the lack of PrEP, studies show that it is primarily a lack of information; doctors did not refer to the high-risk population [24].

In the USA, the spread of pre-exposure prophylaxis programs for HIV prevention is increasing since its approval by the U.S. Food and Drug Administration in 2012, but disparities remain. The main challenge faced now is reducing disparities and including PrEP as an integral part of HIV prevention, sexual health, and primary care [7]. The U.S. PrEP Demonstration Project is a prospective, open-label cohort study assessing PrEP delivery in STD clinics in San Francisco and Miami [25]. The population was MSM and transgender women HIV-uninfected seeking health services in the participant clinics. Participants were offered up to 48 weeks of FCT-TDF for PrEP. The results showed that out of the 921 potentially eligible clients, 60.5% enrolled in the study; 63.5% of the participants admitted to condom-less anal sex in the prior months. Results show interest in PrEp is high among a diverse population of MSM and transgender women at risk for HIV infection when offered in STD and community health clinics. Another study showed the relation between PrEP and decreased levels of HIV anxiety among American MSM [26]. This review got in-depth on how prophylaxis can help men feel safer while having sex with other men. It also stated that FCT-TDF treatment contributes to the decrease in condoms for some populations, raising the concern that education is needed for high-risk populations to understand that PrEP is not a replacement for other methods.

In Florida, the recommendations for PrEP include [27]: Individuals in an ongoing relationship with an HIV-positive partner, Individuals not in a mutually monogamous relationship with a partner who recently tested HIV-negative 'Gay or bisexual men who have anal sex (insertive or receptive) without a condom or have been diagnosed with a sexually transmitted infection (STI) in the past six months,' Heterosexual men or women not regularly using condoms during sex with partners of unknown HIV status, Individuals who inject drugs or have injected illicit drugs in the past six months and who have shared injection equipment or have been in drug treatment for injection drug use in the past six months.

The state of Florida has plans to initiate PrEP for patients without insurance by facilitating free 90 days of FCT-TDF medication after confirming HIV-negative test results for patients. In the case of patients with health insurance, PrEP is typically covered and it is accessible by insurance programs like Medicaid.

Overall, PrEP is an essential tool to fight the scourge of the HIV pandemic. Since there is currently no cure or vaccine, prevention remains the only option to stop HIV infection spread. As reviewed, the underutilization of PrEP treatment among the LGBTQ+ affirming individuals could result from limited providers' knowledge, ineffective communication, and reduced information sharing.

## Conclusions

HIV pre-exposure prophylaxis is currently the best tool, along with the use of condoms, to prevent infection from HIV in high-risk populations. Despite the benefits, PrEP remains an underutilized technique. There seems to be limited education around the benefits of PrEP in most parts of the world. However, it is encouraging to note that about 60% of the high-risk population are willing to consider initiating PrEP medication once they are educated about it. Preventative measures and campaigns about the risk of contracting HIV from high-risk behaviors must continue. At the same time, medical education to medical trainees and students and continuous medication education to physicians should incorporate evidence-based methods of counseling and recommend PrEP to patients that meet the criteria.
